# Diffusion–reaction compartmental models formulated in a continuum mechanics framework: application to COVID-19, mathematical analysis, and numerical study

**DOI:** 10.1007/s00466-020-01888-0

**Published:** 2020-08-13

**Authors:** Alex Viguerie, Alessandro Veneziani, Guillermo Lorenzo, Davide Baroli, Nicole Aretz-Nellesen, Alessia Patton, Thomas E. Yankeelov, Alessandro Reali, Thomas J. R. Hughes, Ferdinando Auricchio

**Affiliations:** 1 Dipartimento di Ingegneria Civile ed Architettura, Università di Pavia, Via Ferrata 3, 27100 Pavia, PV Italy; 2 Department of Mathematics, Emory University, 400 Dowman Drive, Atlanta, GA 30322 USA; 3 Department of Computer Science, Emory University, 400 Dowman Drive, Atlanta, GA 30322 USA; 4 Departments of Biomedical Engineering, Diagnostic Medicine, and Oncology, Livestrong Cancer Institutes, The University of Texas at Austin, 107 W. Dean Keeton St., Austin, TX 78712 USA; 5 Oden Institute for Computational Engineering and Sciences, The University of Texas at Austin, 201 E. 24th Street, Austin, TX 78712-1229 USA; 6 Aachen Institute for Advanced Study in Computational Engineering Science (AICES), RWTH Aachen University, Schinkelstraße 2, 52062 Aachen, Germany

**Keywords:** Epidemic, COVID-19, Compartmental models, Partial differential equations

## Abstract

The outbreak of COVID-19 in 2020 has led to a surge in interest in the research of the mathematical modeling of epidemics. Many of the introduced models are so-called *compartmental models*, in which the total quantities characterizing a certain system may be decomposed into two (or more) species that are distributed into two (or more) homogeneous units called compartments. We propose herein a formulation of compartmental models based on partial differential equations (PDEs) based on concepts familiar to continuum mechanics, interpreting such models in terms of fundamental equations of balance and compatibility, joined by a constitutive relation. We believe that such an interpretation may be useful to aid understanding and interdisciplinary collaboration. We then proceed to focus on a compartmental PDE model of COVID-19 within the newly-introduced framework, beginning with a detailed derivation and explanation. We then analyze the model mathematically, presenting several results concerning its stability and sensitivity to different parameters. We conclude with a series of numerical simulations to support our findings.

## Introduction

Many phenomena in the physical and social sciences feature a *compartmental structure*, in which the total quantities characterizing a system of interest may be decomposed into two (or more) species that are distributed into (two or more) homogeneous units called compartments. As the system evolves in time, the relative distribution of species across the compartments changes, as different physical conditions alter the species state in each compartment and induce species migration from one compartment to another. Compartmental models have been used extensively in biological, ecological, and chemical applications. Notable examples include the *susceptible-infected-recovered* (SIR) models and their variants for epidemic modeling [1–3], the Lotka–Volterra models for predator-prey dynamics [1, 4, 5], pharmacokinetic models used extensively in pharmacology [6], and demographic and migration models found in sociology and demography [7–9].

The majority of compartmental models encountered in the literature consist of systems of ordinary differential equations (ODEs). These models, while simple to formulate, analyze, and solve numerically, are limited in their ability to describe dynamics in both space and time. A common strategy to introduce spatial variation into such ODE models is by defining regional compartments corresponding to different areas in physical space, with coupling terms added to the model equations to account for the movement of species among the different regions [10–13]. This approach was recently employed in [14, 15] to model the spread of COVID-19 among the different administrative regions in Italy. While this approach may be effective for some applications, description of complex spatial dynamics within compartments is difficult and possibly even non-feasible in this framework.

In contrast, compartmental models based on partial differential equations (PDEs) incorporate spatial information more naturally. Specifically, PDE models allow for a space-continuous description of the relevant dynamics, enabling one to describe dynamics in time and space across all scales. This represents a significant advantage over ODE models, whose ability to describe spatial information is limited by the number of spatial compartments one includes. Examples of compartmental models based on PDEs can be found in [16–21]. Likely owing to their apparent increased mathematical complexity and more significant computational burden, compartmental PDE models are less common and, to the authors’ knowledge, a systematic study of compartmental PDE models in a general setting has not been performed.

The present work has two primary goals. First, we aim at formalizing PDE compartmental models in a general framework more familiar to continuum mechanics. Accordingly, we reinterpret such models as fundamental equations of balance and compatibility, with the relationship between the balance and compatibility equations defined by a constitutive relation. We believe that such a framework may be useful to researchers seeking to better understand general compartmental models, and may ultimately help facilitate interdisciplinary collaboration. Our second goal is to improve our understanding of a specific compartmental PDE model, which describes the spatiotemporal spread of COVID-19, and has demonstrated good agreement with the measured data [22], from the physical, mathematical, and numerical points of view. As the reinterpretation of the COVID-19 model within the continuum mechanics framework plays a significant role in this study, the stated goals are complementary.

The current work is organized as follows. We begin by introducing compartmental PDE models within the continuum mechanics framework in Sect. 2. As a preliminary example to aid understanding, we derive a simple two-compartment Lotka–Volterra-type model within this setting. In Sect. 3 we turn our attention to the COVID-19 model discussed in [22], beginning with its derivation within the newly-introduced notational system from continuum mechanics. We analyze the model mathematically and establish its formal sensitivity to diffusivity and its stability in the $L^1$ norm. We also use an ODE variant of the model, which does not incorporate diffusion, to define a basic viral reproduction number $R_0$, which is extensively used as an epidemiological indicator of infectious disease spread. A brief spectral analysis is also performed on the ODE variant. Then, Sect. 4 presents a series of numerical simulations in 1D and 2D to examine different aspects of the COVID-19 model behavior. In 1D, we seek to observe how changes to the spatial and temporal discretization affect the model’s numerical solution. In 2D, we analyze how changes in diffusion affect the physical behavior of the model. For both the 1D and 2D problems, we evaluate the effectiveness of the ODE-derived $R_0$ as a predictor of model behavior, demonstrating the significance of spatial diffusion on modeled viral reproduction. We conclude by summarizing the presented results and suggesting directions for future research in Sect. 5.

## General formulation of compartmental models in a continuum mechanics framework

We consider a system which may be decomposed into *N* distinct species: $u_1(\varvec{x},t),\,u_2(\varvec{x},t),...,u_N(\varvec{x},t)$. Each $u_i$ is a function describing the spatiotemporal distribution of the given species with spatial variable $\varvec{x}$ and time variable *t*. It is often the case that $\sum _{i} u_i$ has a natural interpretation: for example, the $u_i$ may represent well-defined subgroups of a given population, with their sum then yielding the total population. However, this does not always hold. For instance, the $u_i$ may describe the populations of different animal species, rendering their summation physically meaningless without additional normalization. It is always the case, however, that the $u_i$ are the fundamental quantities of interest describing the system dynamics, and change in response to some or all of the other species in the model.

We arrange the $u_i$ in a vector $\varvec{u}$ in $\mathbb {R}^d$ such that $\varvec{u}=[u_1(\varvec{x},t),\,u_2(\varvec{x},t),\ldots ,u_n(\varvec{x},t)]^T$. Rather than using the more traditional notation found in mathematical and biological references, we opt here for a general notational convention more common to continuum mechanics. Hence, over a spatial domain $\Omega$ and a time interval $[t_0,\,t_{end}]$ our equations read:

1 $$\begin{aligned} \partial _t \varvec{u} - \nabla \cdot \varvec{F} + \varvec{b}&= \varvec{0} \end{aligned}$$

2 $$\begin{aligned} \varvec{\varepsilon }&= \nabla \varvec{u} \end{aligned}$$

3 $$\begin{aligned} \varvec{F}&= \varvec{F}(\varvec{u},\varvec{\varepsilon }) \end{aligned}$$

4 $$\begin{aligned} \varvec{b}&= \varvec{b}(\varvec{u}), \end{aligned}$$

plus appropriate initial and boundary conditions. In the system above, Eq. (1) represents a force balance in terms of an internal force $\varvec{F}$, which is thermodynamically conjugate to $\varvec{u}$, and an externally applied force $\varvec{b}$. Physically, we may interpret $\varvec{F}$ as describing the changes in the extensive properties of a given species. Eq. (2) represents the compatibility equation in terms of species $\varvec{u}$ and specie gradient $\varvec{\varepsilon }$. Physically, we may interpret $\varvec{\varepsilon }$ as the specie gradient in space. Then, the relationship between the balance and compatibility equations is defined by the constitutive relation Eq. (3).

The role of the externally applied forces defined in Eq. (4) is fundamental in compartmental models, and warrants some additional discussion. As these forces depend on the unknown variable $\varvec{u}$, often in a nontrivial way, their description as ‘externally applied’ may initially seem inconsistent. To understand why such an interpretation is well-motivated, we recall that Eqs. (1)–(4) describe *N* different species and their relative distribution in time and space. A species $u_a$ may therefore act on a different species $u_b$, such that for $u_b$ this represents an external force. These intra-species interactions are described by $\varvec{b}$ in Eqs. (1) and (4). We note additionally that $\varvec{b}$ may only depend algebraically on $\varvec{u}$. Often, these terms are referred to in the literature as ‘reaction terms’ [17]. We consider Eqs. (1)–(2) to be fundamental and fixed; i.e., any PDE compartmental model will share these equations. The relations in Eqs. (3)–(4) thus define the specific behavior of a given model.

### Example: two-compartment Lotka–Volterra-type model

We illustrate the continuum mechanics framework presented above by first considering a two-compartment *Lotka–Volterra* model, also known as the *predator-prey equations*. This model describes the interaction between two animal populations, predator and prey, in time and space [5]. Let $\varvec{u}= [r,\,w]^T$ where $r(\varvec{x} ,t)$ represents a population of *rabbits* (prey) and $w(\varvec{x} ,t)$ a population of *wolves*.^1^ For ease of dimensional analysis, we denote characteristic population, length and time scales as *P*, *L* and *T*. Our model assumptions are:

1. The movement of both rabbits and wolves exhibit no spatial preference and are independent of each other;
2. The food supply for the rabbits is sufficiently plentiful such that it does not depend on rabbit population (in biological terminology, we say there is no *intraspecific competition* [5]) ;
3. The wolves have no sources of food other than rabbits;
4. The mortality rate of the wolves, as well as the non-predation mortality rate of the rabbits, does not depend on population size.

As the compatibility equation Eq. (2) describes the change in a population resulting from its movement in space, with our constitutive relation Eq. (3) we therefore seek to describe the natural tendency for a given population to move. This tendency to move (or resist movement) can be seen as internal forces that regulate the rate at which movement occurs. Specifically, the source of such forces in the current setting may be the level of exertion required for a member of a population to move a certain distance. Therefore, we consider the following definition for the constitutive relation:^2^

5 $$\begin{aligned} \varvec{F}(\varvec{\varepsilon })&= \varvec{E}\varvec{\varepsilon }, \end{aligned}$$

6 $$\begin{aligned} \varvec{E}&= \begin{bmatrix} {\overline{\nu }}_r &{} 0 \\ 0 &{} {\overline{\nu }}_w \end{bmatrix},\end{aligned}$$

where ${\overline{\nu }}_r>0$ and ${\overline{\nu }}_w>0$ are scalar “diffusion” parameters with units $L^2 T^{-1}$. The line above ${\overline{\nu }}_r$ and ${\overline{\nu }}_w$ is to indicate that these are constant, scalar quantities, a convention we will use throughout the present work. The constitutive relation Eqs. (5)–(6) can also be seen as arising from the limit of a probabilistic random walk [23]. That ${\overline{\nu }}_r$ and ${\overline{\nu }}_w$ are scalars (and not tensors, as may be the case in general) results from assumption 1, which implies that movement exhibits no directional preference [23].

We now define the external forces $\varvec{b}$. Assumption 2 implies that the reproduction rate of rabbits grows with population size without any limiting factor, as their food supply is unconstrained. In mathematical terms, this is expressed as:

7 $$\begin{aligned} \partial _t r \propto {\overline{\alpha }}_r r, \end{aligned}$$

where ${\overline{\alpha }}_r > 0$ is the *reproduction rate* of the rabbits and has units $T^{-1}$.

Assumption 3, however, implies that the reproduction rate of wolves *is* naturally limited by the size of the rabbit population. Accordingly:

8 $$\begin{aligned} \partial _t w \propto \alpha _w (r) w , \end{aligned}$$

with the reproduction rate of the wolves $\alpha _w$ a function of the rabbit population *r*. We consider the simplest possible case and postulate $\alpha _w$ is a linear in *r*:

9 $$\begin{aligned} \partial _t w \propto {\overline{\alpha }}_w r w , \end{aligned}$$

with ${\overline{\alpha }}_w >0$. Note that ${\overline{\alpha }}_w$ has units $T^{-1} P^{-1}$, reflecting its dependence on the local rabbit population. We naturally expect, in turn, that the number of rabbits eaten by wolves increases with the number of wolves. Then,

10 $$\begin{aligned} \partial _t r \propto -\gamma (w)r, \end{aligned}$$

where $\gamma$ is the *predation rate* and depends on *w*. We again assume this function to be linear in *w*, giving:

11 $$\begin{aligned} \partial _t r \propto -{\overline{\gamma }} w r, \end{aligned}$$

where ${\overline{\gamma }} > 0$ has units $T^{-1}P^{-1}$.

Assumption 4 simply states that the mortality of wolves and the mortality of rabbits has no dependence on the population size of either species. Mathematically, we may write this as :

12 $$\begin{aligned} \partial _t r&\propto -{\overline{\mu }}_r r \end{aligned}$$

13 $$\begin{aligned} \partial _t w&\propto -{\overline{\mu }}_w w, \end{aligned}$$

where the *mortality rates* ${\overline{\mu }}_r$ and ${\overline{\mu }}_w$ are both nonnegative and with units $T^{-1}$.

From Eqs. (7)–(13), we may define $\varvec{b}$ as:

14 $$\begin{aligned} \varvec{b}(\varvec{u})&= \varvec{B}\left( \varvec{u}\right) \varvec{u}, \end{aligned}$$

15 $$\begin{aligned} \varvec{B}\left( \varvec{u}\right)&= \begin{bmatrix} -{\overline{\alpha }}_r + {\overline{\mu }}_r &{} {\overline{\gamma }} r \\ -{\overline{\alpha }}_w w &{} {\overline{\mu }}_w \end{bmatrix}.\end{aligned}$$

The relations in Eqs. (5) and (14) are sufficient to define the model in terms of Eqs. (1)–(4). Written in a notation more common to mathematical biology, the model reads:

16 $$\begin{aligned} \partial _t r - \nabla \cdot \left( {\overline{\nu }}_r \nabla r \right) - {\overline{\alpha }}_r r + {\overline{\mu }}_r r + {\overline{\gamma }} w r&= 0 \end{aligned}$$

17 $$\begin{aligned} \partial _t w - \nabla \cdot \left( {\overline{\nu }}_w \nabla w\right) - {\overline{\alpha }}_w r w + {\overline{\mu }}_w w&=0. \end{aligned}$$

## Spatiotemporal model of COVID-19 infection spread

We now discuss the COVID-19 model proposed by Viguerie et al. in [22]. We consider a population *p* of individuals divided into compartments corresponding to disease status, modeling the movement in space and time of the subpopulation in each compartment. Specifically, these compartments are the *susceptible* population *s*, the *exposed* population *e*, the *infected* population *i*, the *recovered* population *r*, and the *deceased* population *d*. Note that *d* refers only to deaths due to COVID-19. We denote the living population pool as $n=s+e+i+r$. Due to the names of the compartments used, this model may be called a *susceptible-exposed-infected-recovered-deceased* (SEIRD) model. We therefore formulate the problem in terms of the vector $\varvec{u}= [s,\,e,\,i,\,r,\,d]^{T}$ containing the different compartments.

### Model derivation and explanation

Following the example of the Lotka–Volterra-type model shown in Sect. 2.1, we begin by making several model assumptions:

1. Movement is proportional to population size; i.e., more movement occurs within heavily populated regions;
2. No movement occurs among the deceased population;
3. There is a latency period between exposure and the development of symptoms;
4. The probability of contagion increases with population size;
5. Some portion of exposed persons never develop symptoms, and move directly from the exposed compartment to the recovered compartment (asymptomatic cases);
6. Both asymptomatic and symptomatic patients are capable of spreading the disease;
7. All living persons are capable of reproduction (the population is not age-structured);
8. The non-COVID-19 mortality rate is independent of the population compartment;
9. New births are susceptible to the virus.

As in the Lotka–Volterra model, the compatibility equation describes the changes in a population due to movement, and the constitutive relation will describe the natural extent to which a given population moves. Assumption 1 above implies that such movement is proportional to the living population size *n*, while assumption 2 sets the movement of the deceased population to zero. Therefore, the constitutive relation for this model is given by:

18 $$\begin{aligned} \varvec{F}&= n \varvec{E} \varvec{\varepsilon }, \end{aligned}$$

19 $$\begin{aligned} \varvec{E}&= \begin{bmatrix} {\overline{\nu }}_s &{} 0 &{} 0 &{} 0 &{} 0 \\ 0 &{} {\overline{\nu }}_e &{} 0 &{} 0 &{} 0 \\ 0 &{} 0 &{} {\overline{\nu }}_i &{} 0 &{} 0 \\ 0 &{} 0 &{} 0 &{} {\overline{\nu }}_r &{} 0 \\ 0 &{} 0 &{} 0 &{} 0 &{} 0 \end{bmatrix}. \end{aligned}$$

Note that the ${\overline{\nu }}_s$, ${\overline{\nu }}_e$, ${\overline{\nu }}_i$ and ${\overline{\nu }}_r$ have units L$^{2}$ T$^{-1}$ P$^{-1}$, in contrast to the units in Eqs. (5)–(6), which were L$^{2}$ T$^{-1}$. This reflects the population-dependent movement rate implied by our assumption 1. Another way to interpret Eqs. (18)–(19) above is as a heterogeneous diffusion process, where the amount of diffusion is proportional to population size.

Having quantified the internal forces with the constitutive relation, we now focus on the external forces. Assumption 3 implies that *all* persons who come into contact with the virus first move to the exposed compartment *e* from the susceptible compartment *s*:

20 $$\begin{aligned} \begin{aligned} \partial _t e \propto -\partial _t s. \end{aligned}\end{aligned}$$

However, assumption 6 implies that this contact could come from both patients showing symptoms (infected population *i*) or patients not showing symptoms (exposed population *e*), and from assumption 4 we conclude that such contact must depend on population size *n*. Therefore,2122Color-coding has been introduced for ease of understanding, to clearly demonstrate that any addition to compartment *e* must be accompanied by an equal subtraction from compartment *s*. We further assume the functions  and  to be linear in *e* and *i* respectively:2324Parameters  and  are called the *contact rates* (units $P^{-1}T^{-1}$) and correspond to the likelihood of contagion resulting from contact with an asymptomatic or symptomatic person, respectively. We now define the function *A*(*n*) as:2526One can naturally see that for $n>> \overline{A}$, the term $\left( 1-\overline{A}/n\right) \approx 1$, increasing with population as desired. $\overline{A}$ is referred to as the Allee parameter (units *P*) and has to be carefully selected [5].

From assumption 3 we know that some portion of the exposed population *e* will become symptomatic after a latency period, and hence move to the infected compartment *i*:2728where  is a parameter corresponding to the latency (or *incubation* period) with units $T^{-1}$. However, from assumption 5, we also know that some portion of the exposed population *e* will never develop symptoms, moving directly to the recovered compartment *r*. These are called *asymptomatic* cases. Therefore:2930where  is the *asymptomatic recovery rate* with units $T^{-1}$. In Eqs. (27)–(30), we see again that subtraction from one compartment is coupled with an equal addition to another.

Some portion of infected patients will recover, leading to movement into the recovered compartment *r*:3132while others will die, moving into the the deceased compartment *d*:3334Parameters  and  are the *symptomatic recovery rate* and *disease mortality rate* respectively, both with units $T^{-1}$. Finally, assumptions 7 and 9 imply that:

35 $$\begin{aligned} \partial _t s \propto {\overline{\alpha }} n, \end{aligned}$$

such that new births enter into the susceptible compartment *s*, with the *birth rate* defined by the parameter ${\overline{\alpha }}$ (units $T^{-1}$). Lastly, assumption 8 states that the deaths that are not due to COVID-19 have no compartmental dependence, implying:

36 $$\begin{aligned} \partial _t s \propto -{\overline{\mu }} s, \end{aligned}$$

with ${\overline{\mu }}>0$ representing the *general mortality rate*, with units $T^{-1}$. Similar terms appear in the exposed, infected, and recovered compartments as well. The terms in Eqs. (35) and (36) are not color-coded because they are not accompanied by a corresponding term of opposite sign in a different compartment.

Finally, Eqs. (25)–(36) allow us to define the external forces for this model as3738We note that the signs in $\varvec{B}$ are reversed when compared to Eqs. (25)–(36), as we have now placed these terms on the external force term $\varvec{b}$ of the left hand side of the equilibrium equation (Eq. (1)).

Additionally, the standard formulation of the COVID-19 model in mathematical biology would be :3940414243

### Mathematical analysis

In this section, we examine four results: the sensitivity equations for the diffusion, the nature of the equilibria of the non-diffusive (space-independent) system, the growth/decay behavior of the total population *n* and the resulting stability in the $L^1$ norm of Eqs. (39)–(43), and a derivation of the basic viral reproduction number $R_0$ for an ODE variant of Eqs. (39)–(43).

#### Sensitivity equations for the diffusion

A fundamental difference between a PDE model such as Eqs. (39)–(43) and an ODE model is the presence of diffusion. Understanding the nature in which the model solution depends on diffusion is therefore of critical importance. To quantify the dependence of the solution on the diffusion parameters ${\overline{\nu }}_s, {\overline{\nu }}_e, {\overline{\nu }}_i, {\overline{\nu }}_r$, we compute the *sensitivity equations*, to determine the quantities

44 $$\begin{aligned} \varvec{s}_\rho \equiv \partial _{\nu _\rho } \varvec{u}, \quad \rho = s,e,i,r. \end{aligned}$$

We proceed by applying standard arguments of perturbation analysis to Eq. (1) using the constitutive relation introduced in Eqs. (18)–(19) and the external forces defined as in Eq. (38). For $\rho = s,e,i,r$ we find the equations:

45 $$\begin{aligned}&\partial _t \varvec{s}_\rho - \nabla \cdot (n \varvec{E}\nabla \varvec{s}_\rho ) -\nabla \cdot ( S_\rho \varvec{E}\nabla \varvec{u}) \nonumber \\&\quad + \varvec{B}(\varvec{u}) \varvec{s}_\rho + [\varvec{u}^T {\mathcal J}^T (\varvec{u})] \varvec{s}_\rho = \nabla \cdot (n\, \mathrm {diag} (\mathbf {e}_\rho ) \nabla \varvec{u}) \end{aligned}$$

where $\mathbf {e}_s = [1,0,0,0,0]$, $\mathbf {e}_e = [0,1,0,0,0]$, $\mathbf {e}_i = [0,0,1,0,0]$, $\mathbf {e}_r = [0,0,0,1,0]$, the third-order tensor ${\mathcal J}$ reads

46 $$\begin{aligned} {\mathcal J}_{ij,k} \equiv \displaystyle {\frac{\partial B_{ij}}{\partial u_k}}, \end{aligned}$$

and $$S_\rho \equiv \sum _{j=1}^5 \varvec{s}_{\rho ,j}$$.

For the sake of notation, set $\Theta (n) \equiv \left( 1 - \frac{\overline{A}}{n} \right)$. Recalling that $n = s + e + i + r$, we have that $\partial _\rho \Theta (n) = - \frac{\overline{A}}{n^2}$ for $\rho = s,e,i,r$. From now on, for simplicity, we set $\Theta ^\prime \equiv - \frac{\overline{A}}{n^2}$.

Notice that the matrix $[\varvec{u}^T {\mathcal J}^T (\varvec{u})]$ has rows from 3 through 5 null since the entries of $\varvec{B}$ are constant. Then, the entries in the rows $i=1,2$ read

$$\begin{aligned}&e \displaystyle {\frac{\partial B_{i2}}{\partial \rho }} \nonumber \\&\quad + i \displaystyle {\frac{\partial B_{i3}}{\partial \rho }} \end{aligned}$$

for $\rho = s,e,i,r$ (in the columns 1,2,3,4), while column 5 is null. This leads to the submatrix47while all the other entries of the $5 \times 5$ matrix $[\varvec{u}^T {\mathcal J}^T (\varvec{u})]$ are 0.

Equations (45) are equipped with homogeneous initial and boundary conditions of the same type of the conditions for $\varvec{u}$. The resulting solution $\varvec{s}$ then describes the sensitivity of a given point in time and space to vary with changes in a given diffusion coefficient.

**Fig. 1:**
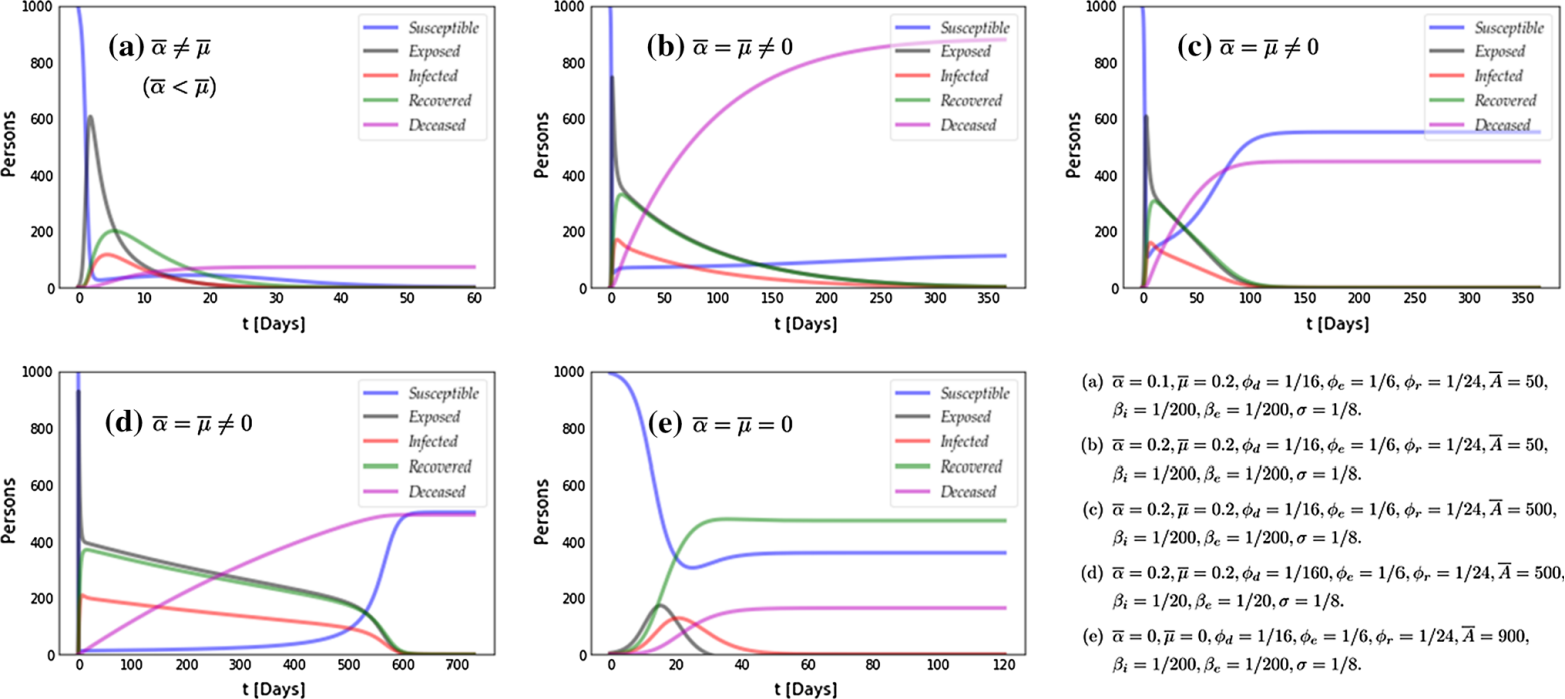
Non-diffusive SEIRD model for different values of the parameters (specified on the right-bottom panel). For all the simulations the initial conditions are $s(0)=994, e(0)=5, i(0)=1, r(0)=d(0)=0$ Persons. The evolution of the solution is consistent with the predictions of our asymptotic analysis

#### Equilibria of the non-diffusive system

An analysis of the equilibria of the non-diffusive (space-independent) system provides guidelines on what to expect for the asymptotic behavior of the solution in time also for our PDE system. The equilibria of the space-independent case are obtained by solving the nonlinear algebraic system

48 $$\begin{aligned} \varvec{B}(\varvec{u}^*) \varvec{u}^* = 0. \end{aligned}$$

It is promptly computed that this system has the following solutions:

- **for** ${\overline{\alpha }}\ne {\overline{\mu }}$ the equilibrium reads $\varvec{u}^* = [0,0,0,0,C_5]$;
- **for** ${\overline{\alpha }}={\overline{\mu }}\ne 0$ the equilibrium reads $\varvec{u}^* = [C_1,0,0,0,C_5]$;
- **for** ${\overline{\alpha }}={\overline{\mu }}=0$ the equilibrium reads $\varvec{u}^* = [C_1,0,0,C_4,C_5]$;

here, $C_1, C_4, C_5$ are constants depending on the initial conditions. Notice that this solution is not incompatible with the occurrence of *n* at the denominator of some entries of $\varvec{B}$, as the singularity is eliminated by the multiplication of the term $\overline{A}/n$ by the terms *se* or *si*.

A local stability analysis around those equilibria can be rapidly achieved, as for $i=0$ the eigenvalues of $-\varvec{B}(\varvec{u}^*)$ are explicitly computed as49These eigenvalues are all real, so we do not expect an oscillatory behavior of the solution in time. If ${\overline{\alpha }} > {\overline{\mu }}$ we have a positive eigenvalue, that means that in absence of diffusion the solution asymptotically diverges in time (as one may expect). For ${\overline{\alpha }} < {\overline{\mu }}$, the equilibrium $\varvec{u}^* = [0,0,0,0,C_5]$ for the first four components is stable.

For ${\overline{\alpha }}={\overline{\mu }}$, we may expect an asymptotic behavior with the susceptible, recovered and deceased converging to a steady nontrivial equilibrium, while exposed and infected tend to the depletion of the epidemic.

Even though this analysis is conducted on a linearized problem, extensive numerical simulations with different values of the parameters show that the results are realistic. In Fig. 1 we report some illustrative results (obtained by a python in-house solver using the NumPy library), probing the asymptotic behavior under different values of the parameters. We tested the case ${\overline{\alpha }} \ne {\overline{\mu }}$ (with ${\overline{\alpha }} < {\overline{\mu }}$ to have a stable equilibrium) in panel (a), the case ${\overline{\alpha }} = {\overline{\mu }} \ne 0$ in panels (b-d) with different values of the parameters and ${\overline{\alpha }} = {\overline{\mu }} = 0$ in panel (e). The specific parameter values for each simulation are also reported in Fig. 1. These values do not represent necessarily realistic scenarios; they are just used to probe the asymptotic behavior of the solution under different conditions. We found the expected equilibria. The comparison among panels (b), (c) and (d) pinpoints the importance of the depensation (Allee) parameter *A* in the pattern of the evolution of the different populations. Depensation impact is particularly evident when *n* becomes small. The accurate identification of the parameters is clearly a major issue for the practical use of these models (see e.g. [24]).

#### Growth/decay of the total population and $L^1$ stability

In this section, we examine the behavior of Eqs. (39)–(42). One easily observes from the lack of diffusion in Eq. (43) and final column of $\varvec{B}(\varvec{u})$ in Eq. (38) that *d* does not influence the dynamics of the system. Adding Eqs. (39)–(42) together, we observe cancellation of all the colored terms except , leaving:50We let $\eta _1 = s$, $\eta _2=e$, $\eta _3=i$, $\eta _4=r$, ${\overline{\nu }}_1={\overline{\nu }}_s$, ${\overline{\nu }}_2={\overline{\nu }}_e$, ${\overline{\nu }}_3={\overline{\nu }}_i$, ${\overline{\nu }}_4={\overline{\nu }}_r$ and rewrite Eq. (50), yielding:51We now multiply Eq. (51) by a test function *w* and integrate over $\Omega$, giving:52Applying the divergence theorem, we obtain53From the assumption of zero-flux boundary conditions, the boundary term on the right-hand side of Eq. (53) vanishes. Note that $\varvec{n}$ is the outward-pointing normal vector on $\partial \Omega$ and is not to be confused with *n*. We now let $w=1$ globally, causing the third term on the right-hand side of Eq. (53) to vanish as well, leaving:54We define the *total population* *N*(*t*) and *total infected population* *I*(*t*) as:

55 $$\begin{aligned} N(t)&= \int _{\Omega } n(\varvec{x},t) \, \partial \Omega \quad \text {and } \end{aligned}$$

56 $$\begin{aligned} I(t)&= \int _{\Omega } i(\varvec{x},t)\, \partial \Omega \end{aligned}$$

respectively. Then, Eq. (54) can be rewritten as the ODE:57whose solution is:58which describes the growth/decay behavior of the total population *N*. This also amounts to an $L^1$ stability result for the system, assuming $s,\,e,\,i,\,r>0$ (as is the case in the present applications).

##### Remark

If one assumes that the diffusivities are constant and all equal, Eq. (50) reduces further to:59The above suggests that one may interpret the global behavior of the system as a nonlinear continuity equation for *n* transported over the convective field $\nu \nabla n$. It can also be interpreted as a reaction–diffusion equation.

#### Determination of $R_0$

The basic viral reproduction number $R_0$ serves an important role in the discussion of SIR-type models. In a wholly susceptible population, $R_0$ describes the average number of additional infections caused by each infected individual. Naturally, $R_0>1$ implies growth of the epidemic, whereas $R_0<1$ implies decay in infectious spread [5]. The concept of $R_0$ is well-defined for ODE models. However, its extension to a PDE model is unclear, owing to the influence of diffusion. We derive $R_0$ for the ODE version of the PDE model given by Eqs. (39)–(43) and will evaluate its efficacy with numerical tests in Sect. 4.

The ODE version of the COVID-19 model reads:6061626364Here, we denote the time derivatives with dots, as we now consider the derivative of a function of a single variable, rather than partial derivatives as done previously in this work. For simplicity, we are not considering non-COVID19 deaths, new births, and the Allee term (hence, ${\overline{\mu }} = {\overline{\alpha }}=\overline{A}=0$; although their inclusion is not a problem for the analysis shown here.

We proceed using the *next-generation matrix* procedure outlined in [25]. This approach considers all compartments regarded as ‘diseased’ in a given model. ‘Diseased’ in this context means groups capable of transmitting the infection to others. The terms in the model corresponding to new diseased cases are grouped into a matrix $\varvec{N}$, while the terms describing the movement of existing diseased cases into different compartments are grouped into a matrix $\varvec{V}$. The basic reproduction number $R_0$ is then obtained as the spectral radius of $\varvec{N}\varvec{V}^{-1}$. The justification for this is based on the Perron-Frobenius theorem and is not straightforward. The interested reader is referred to [25].

In our model, there are two compartments that we consider ‘diseased’: the *exposed* and the *infected* compartments. Thus, we consider the equation:

65 $$\begin{aligned} \begin{aligned} \begin{bmatrix} \dot{e}\\ \dot{i} \end{bmatrix}&= \left( \varvec{N} - \varvec{V} \right) \begin{bmatrix} e \\ i \end{bmatrix}. \end{aligned}\end{aligned}$$

As stated above, $\varvec{N}$ is the matrix containing the new appearances of diseased patients into any compartment, and $\varvec{V}$ contains terms which transfer already diseased individuals from one compartment to another. In this case, we stress that movement from *e* to *i* is due to the matrix $\varvec{V}$, as an exposed patient moving to the infected category is not considered a new entry into the ’diseased’ category and hence does not participate in $\varvec{N}$. Thus, we define $\varvec{N}$ and $\varvec{V}$ as:66A simple computation shows:67which in turn yields:68Hence,69Applied directly to the ODE model Eqs. (60)–(64), the above Eq. (69) will provide an indication of viral growth rate, as intended. However, given that Eq. (69) does not account for the diffusion present in Eqs. (39)–(43), its effectiveness as an indicator of viral reproduction for the PDE model is unclear and will be examined during the numerical simulations.

## Numerical simulations

In this section, we present two numerical simulation studies of the COVID-19 model in 1D and 2D, respectively, to examine the behavior of the model in Eqs. (39)–(43) in detail.

### 1D simulation study

In this section, we perform a series of simulations using a one-dimensional version of the model in Eqs. (39)–(43). We aim at examining the impact of various numerical solution techniques. In particular, we analyze the spatial and temporal convergence of the computed solutions over various discretization schemes. We also examine the model dynamics more generally and evaluate the efficacy of the $R_0$ definition Eq.  (69) for the PDE model.

#### Problem setup

We consider the spatial domain $\Omega$ given by [0, *L*] and a time interval [0, *T*], with $T=200$ days. In the simulations presented in this section, we normalize in space with respect to the characteristic length *L* of the spatial domain. Hence, we denote $x^*=x/L$. The domain is populated with a population distribution with the unit “Persons.” One may interpret it as denoting a generic normalized population, as we have done with the length scale. The units and values for the relevant space-normalized parameters for the simulations are accordingly presented in Table 1.

**Table 1:** Parameter values for the 1D simulations

Parameter	Units	Value
	$\hbox {Days}^{-1}$	1/8
	$\hbox {Persons}^{-1} \cdot$ $\hbox {Days}^{-1}$	1/2
	$\hbox {Persons}^{-1} \cdot$ $\hbox {Days}^{-1}$	1/2
	$\hbox {Days}^{-1}$	1/24
	$\hbox {Days}^{-1}$	1/6
	$\hbox {Days}^{-1}$	1/160
$\mu$	$\hbox {Days}^{-1}$	0
$\alpha$	$\hbox {Days}^{-1}$	0
${\overline{\nu }}_s^*$	$\hbox {Persons}^{-1} \cdot$ Days $^{-1}$	5$\cdot 10^{-5}$
${\overline{\nu }}_e^*$	$\hbox {Persons}^{-1} \cdot$ Days $^{-1}$	1$\cdot 10^{-3}$
${\overline{\nu }}_i^*$	$\hbox {Persons}^{-1} \cdot$ Days $^{-1}$	1$\cdot 10^{-10}$
${\overline{\nu }}_r^*$	$\hbox {Persons}^{-1} \cdot$ Days $^{-1}$	5$\cdot 10^{-5}$

Note all values have been normalized in space by a characteristic length scale *L*, with this normalization reflected in the units

For the initial conditions, we set $s(x^*,0) = s_0(x^*)$ and $e(x^*,0)=e_0(x^*)$ as follows

70 $$\begin{aligned} s_0(x^*)&= e^{-(x^*+1)^4} + e^{-\frac{(x^*-.35)^2}{1e-2}} + \frac{1}{8}\left( e^{-\frac{(x^*-.62)^4}{1e-5}} \right. \nonumber \\&\quad \left. + e^{-\frac{(x^*-.52)^4}{1e-5}} + e^{-\frac{(x^*-.42)^4}{1e-5}}\right) + \frac{1}{4} e^{-\frac{(x^*-.735)^4}{1e-5}}, \end{aligned}$$

71 $$\begin{aligned} e_0(x^*)&= \frac{1}{20}e^{-\frac{(x^*-.75)^4}{1e-5}}. \end{aligned}$$

**Fig. 2:**
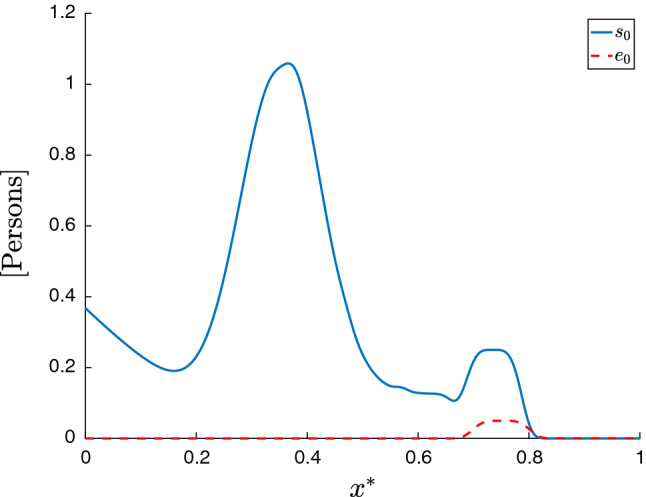
Initial values for susceptible compartment $s_0$ and exposed compartment $e_0$ for the 1D simulations

Figure 2 shows these initial conditions. We further set $i(x^*,0)=0$, $r(x^*,0)=0$, and $d(x^*,0)=0$. Qualitatively, these initial conditions represent a large population center around $x^*=.35$ with no exposed persons and a small population center around $x^*=.75$ with some exposed individuals. We also enforce homogeneous Neumann boundary conditions at $x^*=0$ and a zero-population Dirichlet boundary condition at $x^*=1$ for all model compartments. The latter represents a non-populated area at $x^*=1$.

Additionally, to assess mesh and time integration convergence, we will analyze the total infected population *I*(*t*), defined previously as Eq. (56), and the analogously-defined total deceased population *D*(*t*). We will also study the time evolution of the total susceptible population *S*(*t*), the total exposed population *E*(*t*) and the total recovered population *R*(*t*), all defined analogously to *I*(*t*).

#### Numerical methods

We use linear finite elements to discretize the spatial domain and we integrate in time using either a second-order implicit (BDF2) or first-order implicit Backward Euler scheme. Each time step is solved fully implicitly using a Picard linearization. All linear systems are solved using GMRES with a Jacobi preconditioner. We employ mass-lumping on all reaction terms.

#### Mesh convergence

In this analysis, we compare numerical solutions computed on successively refined uniform grids with mesh size $\Delta x^*$=1/500, 1/1000, 1/2000, and 1/4000. Time integration in this study is performed exclusively with a BDF2 scheme using a constant time step $\Delta t=0.25$ days.

In Table 2, we assess mesh convergence using the peak infection date $\hat{t}$, the peak total infected population $I(t^*)$, and the final total deceased population *D*(*T*). As the peak infection date for $\Delta x^*=$ 1/4000 is $\hat{t}=$118 days, we also evaluate *I*(118) for each level of spatial resolution. We observe a steady increase in all these metrics as $\Delta x^*$ is refined and they all progressively approach the corresponding result for the finest mesh. In particular, the quantities reported in Table 2 vary less than 1% between $\Delta x^*$=1/2000 and $\Delta x^*$=1/4000, which suggests a good level of spatial convergence for $\Delta x^*$=1/2000.

**Table 2:** Mesh convergence of 1D simulations in terms of the peak infection date $\hat{t}$, the peak total infected population $I(\hat{t})$, the total infected population at peak date of the finest mesh *I*(118) , and the final total deceased population *D*(*T*)

$\Delta x^*$	$\hat{t}$	$I(\hat{t})$	*I*(118)	*D*(*T*)
1/500	122	.038401	.037923	.01265
1/1000	119	.038556	.038482	.012804
1/2000	119	.038667	.038662	.012875
1/4000	118	.038738	.038738	.012910

The relative difference of all these metrics between the cases $\Delta x^*$=1/2000 and $\Delta x^*$=1/4000 is inferior to 1%

**Fig. 3:**
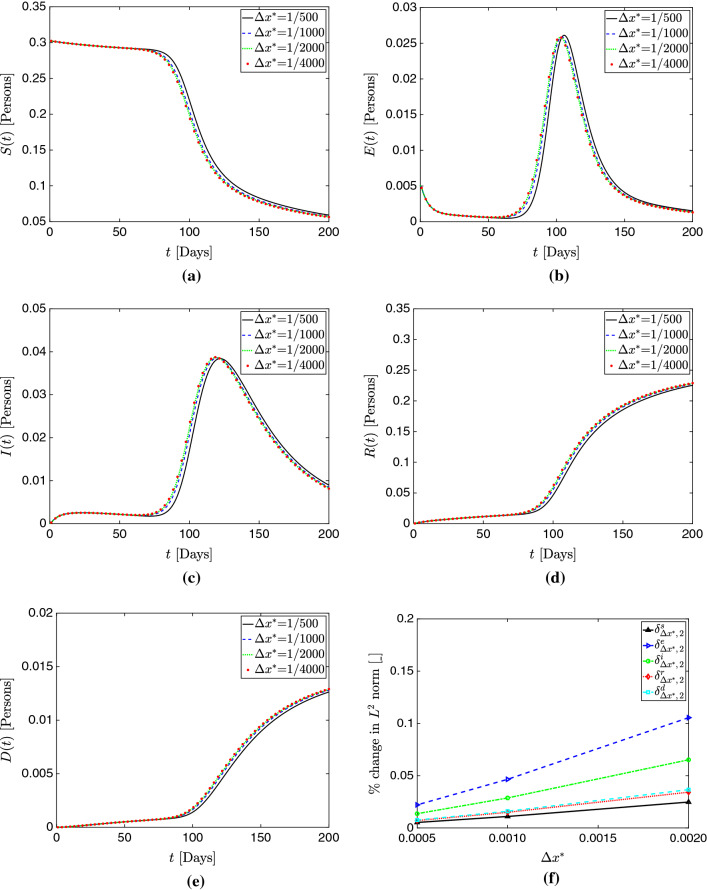
Mesh convergence analysis in the 1D simulation study. **a** Total susceptible population *S*(*t*). **b** Total exposed population *E*(*t*). **c** Total infected population *I*(*t*) . **d** Total recovered population *R*(*t*). **e** Total deceased population *D*(*t*). **f** Percent change in $L^2$ norm with successive refinement. These plots show evidence of mesh convergence, with the solutions for $\Delta x^*$=1/2000 and $\Delta x^*$=1/4000 showing minimal differences

**Fig. 4:**
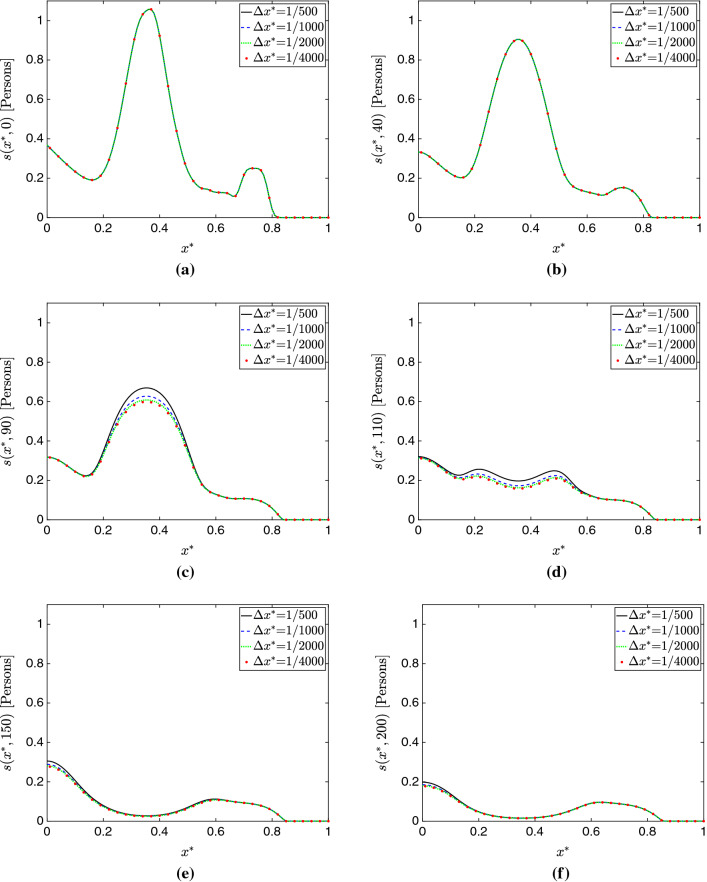
Evolution of the susceptible population compartment $s(x^*,t)$ over time for varying mesh sizes in 1D. **a** $t=0$ days. **b** $t=40$ days. **c** $t=90$ days. **d** $t=110$ days. **e** $t=150$ days. **f** $t=200$ days. We see similar results across the different meshes, with some noticeable transient discrepancy occurring at *t*=90 and *t*=110 days. This indicates that the coarser mesh resolutions cause *dispersion error*, in which the phase of the solution is affected. In this instance, the solution on the coarse meshes appears delayed

**Fig. 5:**
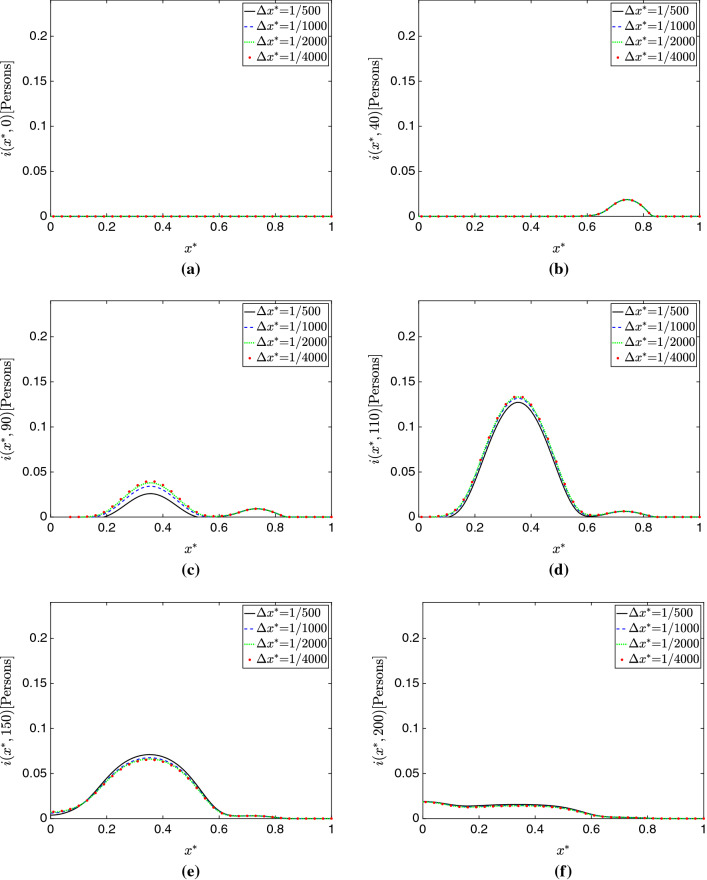
Evolution of the infected population compartment $i(x^*,t)$ over time for varying mesh sizes in 1D. **a** $t=0$ days. **b** $t=40$ days. **c** $t=90$ days. **d** $t=110$ days. **e** $t=150$ days. **f** $t=200$ days. We see noticeable transient discrepancy occurring at *t*=90, *t*=110, and $t=150$ days, again suggesting dispersion error arising from the coarse discretizations

**Fig. 6:**
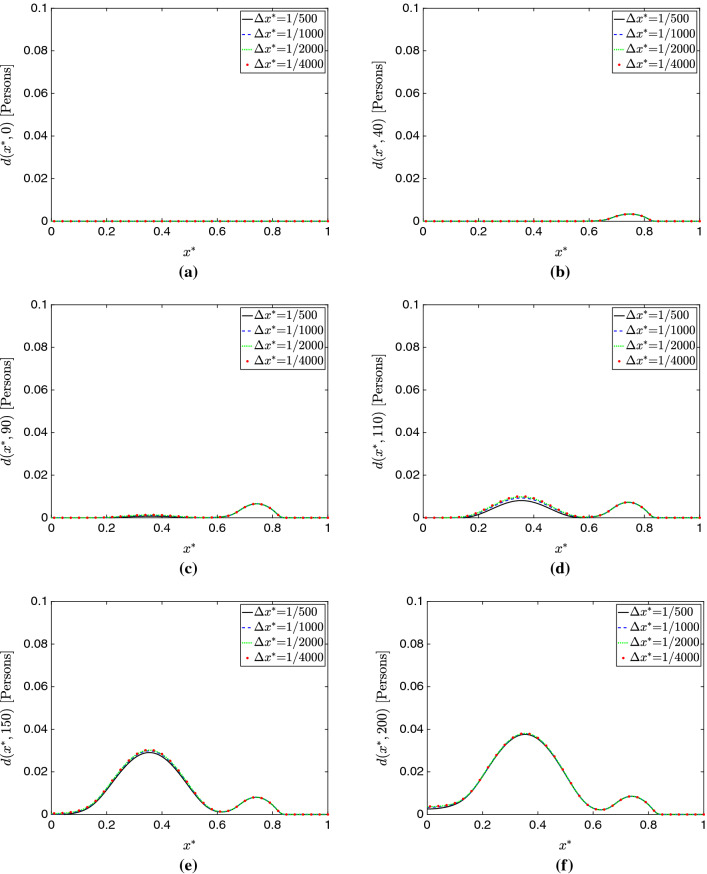
Evolution of the deceased population compartment $d(x^*,t)$ over time for varying mesh sizes in 1D. **a** $t=0$ days. **b** $t=40$ days. **c** $t=90$ days. **d** $t=110$ days. **e** $t=150$ days. **f** $t=200$ days. We see similar results across the different meshes, with some noticeable transient discrepancy occurring at *t*=90 and *t*=110 days, where once again the dispersion error on the coarse meshes is apparent

Figure 3a–e show plots of the total populations *S*(*t*), *E*(*t*), *I*(*t*), *R*(*t*), and *D*(*t*) for all the mesh sizes considered in this study. Additionally, Figs. 4, 5, and 6 respectively present plots of *s*(*x*, *t*), *i*(*x*, *t*) and *d*(*x*, *t*) for the different spatial resolutions. Qualitatively, these plots confirm the existence of mesh convergence, as the difference in the plotted variables progressively reduces as we refine the mesh. Indeed, the change between the results for $\Delta x^*=1/2000$ and $\Delta x^*=1/4000$ cases is negligible.

We further assess mesh convergence with an operator $\delta _{\Delta x^*,\,r}^c$ that evaluates the percent change in $L^2$ norm for each model compartment *c* when a given mesh resolution $\Delta x^*$ is refined by a factor of *r*:

72 $$\begin{aligned} \delta _{\Delta x^*,\,r}^c&= \sqrt{\frac{\displaystyle \int _{0}^{T}\left( \displaystyle \int _0^1 c_{\Delta x^*/r} (x^*,t)- c_{\Delta x^*} (x^*,t)\,dx^* \right) ^2 \, dt}{ \displaystyle \int _{0}^{T} \left( \displaystyle \int _0^1 c_{\Delta x^*/r} (x^*,t)\, dx^* \right) ^2 \,dt }}. \end{aligned}$$

In Fig. 3f, we plot the values of this operator for all compartments and $\Delta x^*$=1/500, 1/1000, 1/2000 (note the refinement ratio *r*=2 for all cases). Again, we observe good evidence of mesh convergence, as $\delta _{1/2000,2}^c$ is notably smaller than $\delta _{1/500,2}^c$ and $\delta _{1/1000,2}^c$ for all compartments *c* in the model.

An interesting phenomenon we observe is that the largest source of error does *not* seem to come from over-diffusion or an underestimation of peaks. In fact, peak quantities are predicted similarly across schemes with only slight variation; instead, *dispersion error*, in which the primary source of error is not the magnitude but instead the *phase* of the solution, seems the largest problem here. This is particularly apparent looking at Fig. 3, where the cases of $\Delta x^*=1/500$ appear similar to the more refined simulations, but with a delay in their occurrence. This is further supported by the predictions of $\hat{t}$ shown in Table 2. Referring to Figs. 4c, d, 5c, d, and 6c, d, one may see this effect in time across various compartments.

**Fig. 7:**
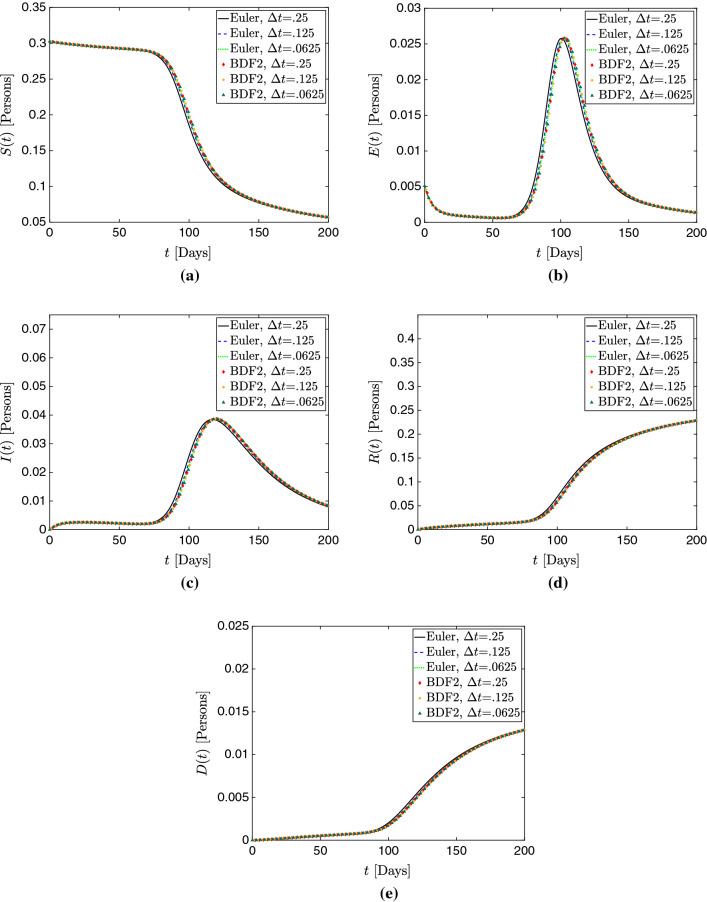
Temporal convergence analysis in the 1D simulation study. **a** Total susceptible population *S*(*t*). **b** Total exposed population *E*(*t*). **c** Total infected population *I*(*t*) . **d** Total recovered population *R*(*t*). **e** Total deceased population *D*(*t*). The model solutions obtained with the Backward Euler method change appreciably when the time step is reduced. In contrast, the BDF2 solutions appear well-resolved in time and change minimally as we refine the time step

**Table 3:** Temporal convergence of 1D simulations in terms of the peak infection date $\hat{t}$, the peak total infected population $I(\hat{t})$, and the final total deceased population *D*(*T*)

$\Delta t$	Scheme	$\hat{t}$	$I(\hat{t})$	*D*(*T*)
0.25	Backward Euler	116	.0384732	.0129437
0.125	Backward Euler	118	.0385545	.0129038
0.0625	Backward Euler	118	.0386006	.0128836
0.25	BDF2	119	.0386668	.0128755
0.125	BDF2	119	.0386587	.0128688
0.0625	BDF2	119	.0386536	.0128658

As we reduce $\Delta t$, the selected metrics show a slight variation for the Backward Euler method, while the changes are negligible for the BDF2 scheme

#### Temporal convergence

In this analysis, we examine the impact of time integration and time-step size $\Delta t$ on the numerical approximation of the model solution. We consider both the Backward Euler and BDF2 time integration schemes with time step sizes $\Delta t$=0.25, 0.125, and 0.0625 days. As the results in Sect. 4.1.3 suggested $\Delta x^*$=1/2000 was a sufficiently fine spatial discretization, we utilize this mesh resolution here. Table 3 reports the peak infection day $\hat{t}$, the peak total infection population $I(\hat{t})$, and final total deceased population *D*(*T*) for each $\Delta t$ and time integration scheme. As we reduce $\Delta t$, these quantities slightly vary for the Backward Euler scheme, while the changes are negligible for the BDF2 schemes. Additionally, we plot the time evolution of the total population in each model compartment in Fig. 7 for all time steps considered in this analysis and for both time integration algorithms. These plots also show that the results for the Backward Euler method exhibit small but perceptible difference, while the solutions obtained with the BDF2 scheme are virtually the same for all time steps.

We also define relevant error quantities for a compartment *c* using a related but distinct notation to Eq. (72). Some adjustments must be made owing to the fact that we now consider not only one point of comparison as before (the spatial resolution resolution in the case of Eq. (72)) but two (both time step size and time integration scheme). For a compartment *c*, this quantity reads:

73 $$\begin{aligned} \delta _{\Delta t, SCHEME}^c \end{aligned}$$

where *c* gives the compartment, $\Delta t$ the time step, and *SCHEME* the time integration scheme. In all instances, we compare with the case computed using BDF2 with $\Delta t=.0625$. So, for example, to quantify relative error of the solution of *c* computed with the Backward-Euler scheme (BE) using a given $\Delta t$, $\delta ^c_{\Delta t,\,BE}$ is defined as:

74 $$\begin{aligned}&\delta _{\Delta t,\,BE}^{c} \nonumber \\&\quad = \sqrt{\frac{\displaystyle \int _{0}^{T}\left( \displaystyle \int _0^1 c_{\Delta t,\,BE} (x^*,t)- c_{.0625,\,BDF2} (x^*,t)\,dx^* \right) ^2 \, dt}{ \displaystyle \int _{0}^{T} \left( \displaystyle \int _0^1 c_{.0625,\,BDF2} (x^*,t)\, dx^* \right) ^2 \,dt }}. \end{aligned}$$

**Fig. 8:**
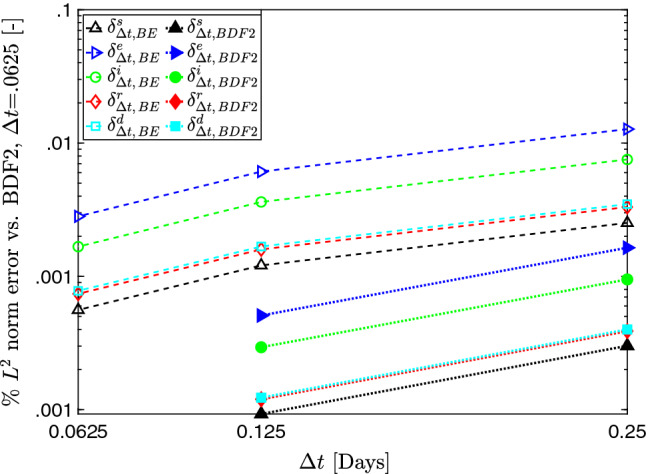
Percent difference in the $L^2$ norm between 1D solutions obtained with the Backward Euler (dashed lines) and BDF2 methods (dotted lines) for each $\Delta t$. All cases are compared to the BDF2 solution with $\Delta t=.0625$, with the formal of definition $\delta$ in Eq. (74). The decreasing trends in both plots show temporal convergence. The BDF2 appears well-resolved in time for even the coarsest time step $\Delta t$=.25 days. The Backward Euler method requires a fine time step to render results with comparable accuracy to the BDF2 scheme.

Figure 8 plots the temporal convergence in terms of Eq. (74). We observe that the $L^2$ norm difference decreases as $\Delta t$ is refined for all model compartments, which indicates temporal convergence. The solutions obtained with the Backward Euler method differ noticeably at the coarser time steps, but this difference reduces as we refine $\Delta t$. In contrast, BDF2 appears to be well-resolved in time even at the coarsest time step $\Delta t=.25$, with the refinement to $\Delta t=.125$ showing minimal decrease in $L^2$ norm. Thus, the BDF2 scheme provides satisfactory time resolution, even for large time steps.

**Fig. 9:**
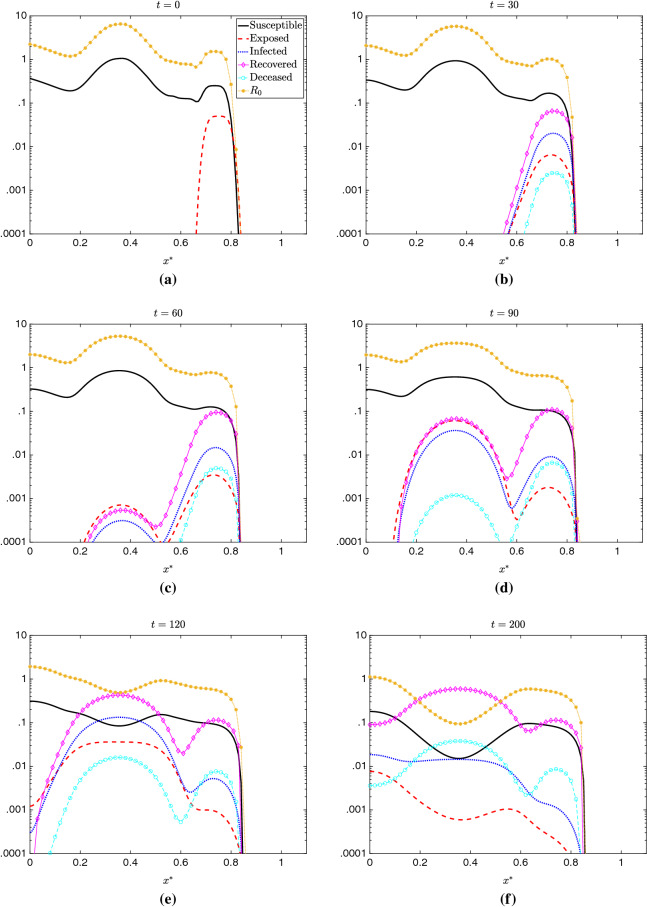
Evolution of all model compartments and $R_0$ as defined by Eq. (69) in time and space in 1D. At $t=0$ days (**a**), we see an initial exposed population centered around $x^*=.75$. As time progresses to $t=30$ days (**b**), the outbreak around $x^*=.75$ has grown, with increasing numbers of infected, recovered, and deceased individuals in that region. By $t=60$ days (**c**), we begin to see the infection reach the large population center around $x^*=.35$, and by $t=90$ days (**d**), the outbreak severity in the areas around $x^*=.35$ and $x^*=.75$ are similar. By $t=120$ days, the outbreak around $x^*=.75$ has died down, with the area around $x^*=.35$ now the most affected region; owever, the $R_0<1$ around $x^*=.35$ indicates that the epidemic may begin to subside. This is indeed the case, and by $t=200$ days (**f**), we see decreases in infections and increases in recoveries near $x^*=.35$

#### Model dynamics

This analysis focuses on the general model behavior, which we examine in a simulation using the BDF2 scheme with $\Delta t$=.0625 days, $\Delta x^*$=1/2000. The results for all model compartments are shown in Fig. 9. The infection begins localized in a small population center around $x^*=.75$ and remains localized for the first part of the simulation. At day $t\approx 60$, the virus reaches the large population center at $x^*=.35$, and the number of infections begins to increase dramatically. By day $t=200$, nearly all of the population near $x^*=.35$ has been exposed to the virus. Eventually, due to lack of susceptible individuals, the virus spread ceases.

**Fig. 10:**
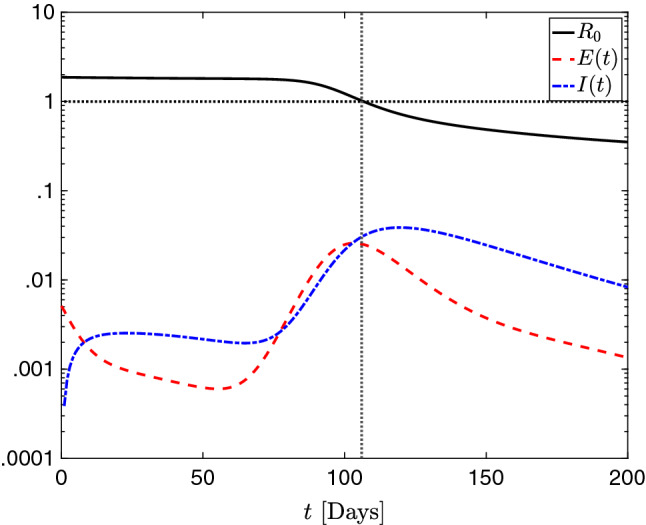
Evolution in time of $R_0$ as defined by Eq. (69) as well as the total exposed and total infected populations in 1D. We see that $R_0$ is in good agreement with the observed model dynamics, with the decrease of new exposures corresponding nearly exactly to the point where $R_0<1$ (indicated with the dotted horizontal and vertical lines for ease of visualization). The presence of diffusion, not accounted for in Eq. (69), is likely the source of the slight discrepancy

In Fig. 10, we compare $R_0$ as defined by Eq. (69) with the exposed and infected compartments. Although the definition in Eq. (69) does not account for diffusion, we observe that $R_0$ still predicts model behavior reasonably well, with the point where $R_0<1$ corresponding almost exactly with the decrease in new exposures. This is further corroborated by the results depicted in Fig. 9, where the regions where $R_0<1$ at $t=0$ ultimately show very little contagion, and indeed a distinct ‘hitch’ forms in the distribution infections between the two population centers. Although there is some slight discrepancy owing to the diffusion, we find the definition of $R_0$ given by (69) to be a reliable predictor of the viral behavior for this 1D simulation scenario.

The model dynamics shown in the 1D simulation in Fig. 9 is similar to that shown for Lombardy in [22] and in the following section. Indeed, for sustained spread of the disease, a certain level of population density is required. Although the disease contagion will diffuse through low-density regions, the growth in those areas tends to be small. Though there have been some notable exceptions, this behavior pattern is similar to what has been observed worldwide, where low population-density regions have largely avoided the catastrophic contagion found in high-density areas [26] .

### 2D simulation study

The primary difference between the PDE version and the ODE version of the COVID-19 model lies in the influence of the diffusive term. The impact of diffusion on disease spread is *a priori* difficult to quantify. Increased diffusion leads to a faster and wider dispersion of the virus. However, it also has regularizing effects and may reduce peaks in general. Therefore, exploring such dynamics in detail is important for a full understanding of the model.

In this section, we examine the role of diffusion using the Italian region of Lombardy as our test geometry, using both qualitative analysis and the formally derived sensitivity equation shown in Eq. (45). The problem configuration is identical to the one given in [22] for the simulation scenario labelled ‘Global Reopening B’. This simulation is intended to model the spread of the COVID-19 epidemic in Lombardy, beginning on February 27, accounting for various governmental restrictions and relaxations as they occur. We report the relevant parameter values in Table 4. Good agreement between the presented simulation setup and measured data was shown in [22], and we refer the reader to [22] for a detailed comparison of simulated and measured data. The problem was solved using linear finite elements on an unstructured triangular mesh. The time integration was performed with a Backward Euler scheme, with a Picard-type linearization used to solve the nonlinear system at each time step. All linear systems were solved with GMRES using a Jacobi preconditioner.

In addition to the simulation shown in [22], we now examine two additional cases: one in which the values of ${\overline{\nu }}_s$, ${\overline{\nu }}_e$, ${\overline{\nu }}_i$ and ${\overline{\nu }}_r$ are doubled, and another in which they are halved. We also consider a case in which ${\overline{\nu }}_r$, ${\overline{\nu }}_e$, and ${\overline{\nu }}_i$ are doubled but ${\overline{\nu }}_s$ is halved. This is similar to the parameter setup in the 1D simulations. The main motivation is to avoid possibly nonphysical diffusion among the susceptible population, causing reduced population density in general.

**Fig. 11:**
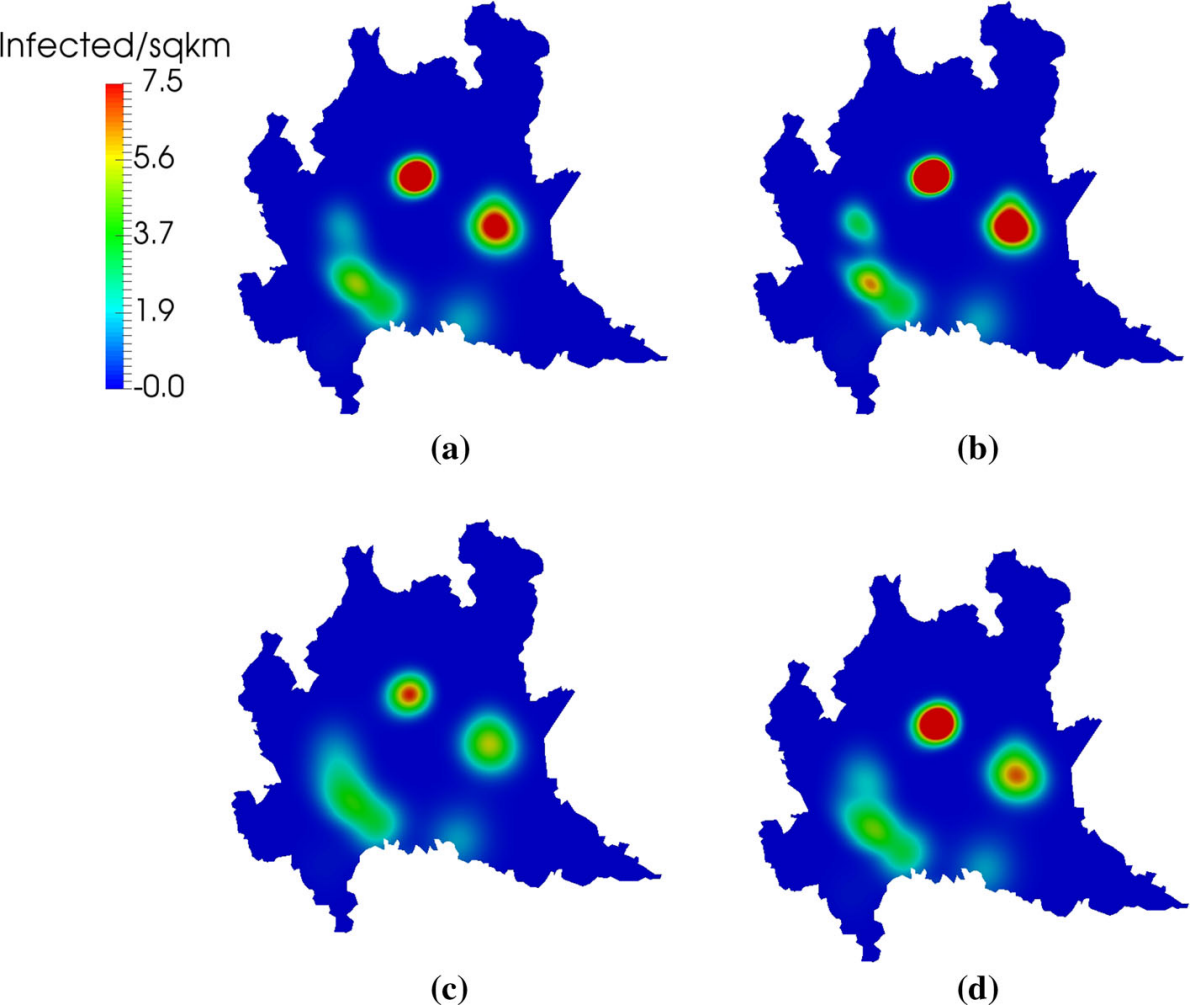
Spatial distribution of the infected population at $t=14$ days in the 2D simulations over the Italian region of Lombardy. **a** Baseline scenario. **b** Half-diffusion case. **c** Double-diffusion case. **d** Simulation case in which the baseline ${\overline{\nu }}_r$, ${\overline{\nu }}_e$, and ${\overline{\nu }}_i$ are doubled but ${\overline{\nu }}_s$ is halved. With halved diffusion (**b**), we see that outbreaks are more severe, but also concentrated in smaller regions, particularly apparent in the southwest. In contrast, increased diffusion (**c**) show a less intense peak over a greater overall area. In **d**, where the diffusion among susceptibles is decreased but increased in other compartments, outbreak severity seems similar to the baseline in **a**, but covering a slightly larger area (again, most apparent in the southwest)

**Fig. 12:**
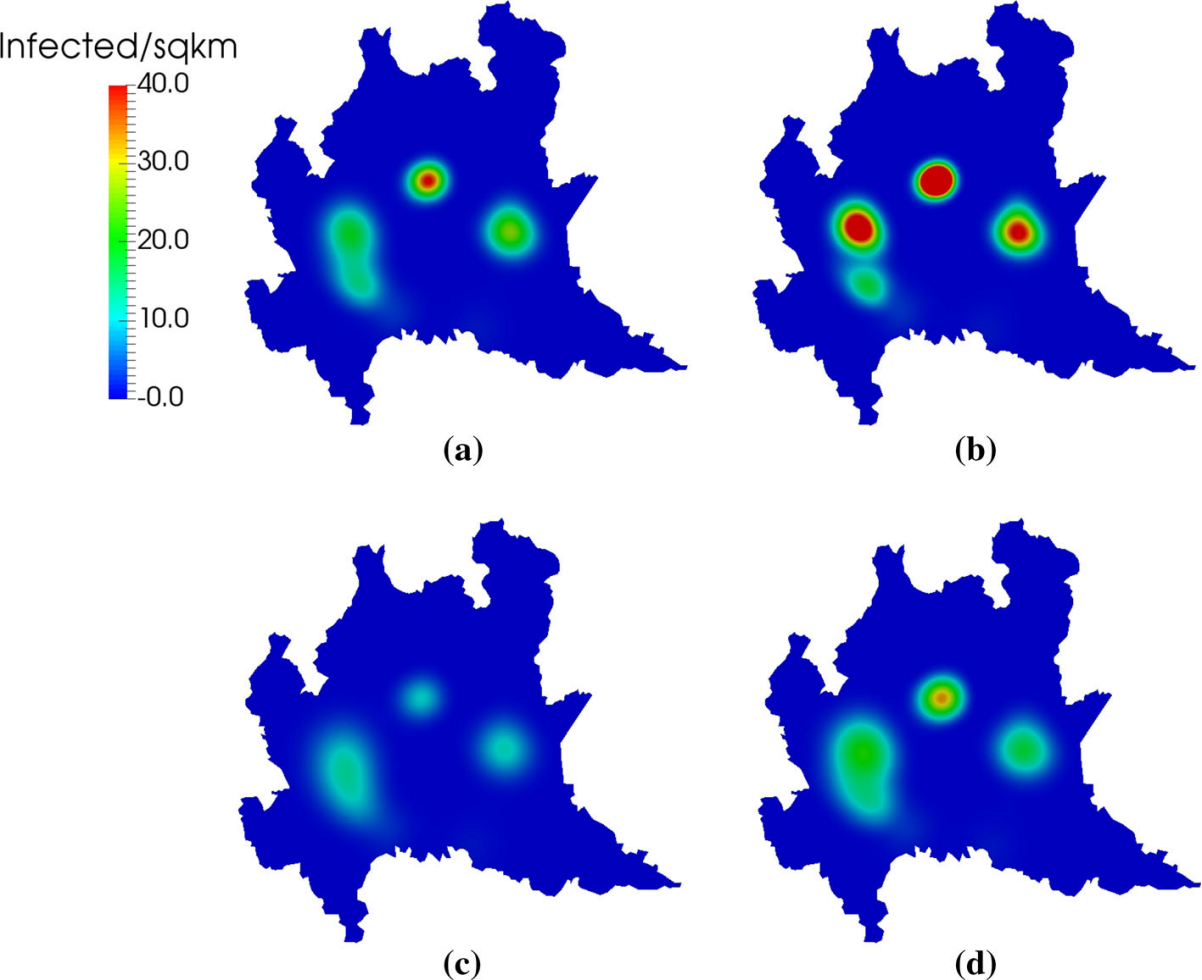
Spatial distribution of the infected population at $t=30$ days in the 2D simulations over the Italian region of Lombardy. **a** Baseline scenario. **b** Half-diffusion case. **c** Double-diffusion case. **d** Simulation case in which the baseline ${\overline{\nu }}_r$, ${\overline{\nu }}_e$, and ${\overline{\nu }}_i$ are doubled but ${\overline{\nu }}_s$ is halved. In **b**, we see both increased severity and interesting localization dynamics; in **a**, **c**, and **d** there appear to be three primary epicenters of infection, while in the case of **b** there appear to be four. The outbreak in **c** is much less severe than the other cases, owing to the increased diffusion. In the case of **d**, we see a larger overall infected area and similar intensity of infection to the baseline (**a**)

Figures 11 and 12 show the spatial distribution of infected individuals at *t*=14 and *t*=30 days, respectively. We see that larger diffusion leads to a wider geographic range of affected areas. This is particularly noticeable in the southeastern clusters in Fig. 11. There, the double-diffusion case produces a homogeneous, continuous region of infection. In contrast, the half-diffusion case shows more localized dynamics, and a clear separation into distinct regions. This separation is maintained in Fig. 12 at *t*=30 days, whereas the baseline and double-diffusion cases predict a single, larger area of infection. The simulation case in which ${\overline{\nu }}_r$, ${\overline{\nu }}_e$, and ${\overline{\nu }}_i$ are doubled but ${\overline{\nu }}_s$ is halved produces intermediate results between the double-diffusion and half-diffusion cases. In all cases, we note that the outbreak path follows regions of high population density, which is the expected behavior given the constitutive relation defined by Eqs. (18)–(19).

**Table 4:** Parameter values for the 2D Lombardy simulations

Parameter	Units	Feb.27-Mar.9	Mar.9-22	Mar.22-28	Mar.28-May3	May3-
	$\hbox {Days}^{-1}$	1/7	1/7	1/7	1/7	1/7
	$\hbox {Persons}^{-1}\cdot$ $\hbox {Days}^{-1}$	3.3$\cdot 10^{-4}$	8.5$\cdot 10^{-5}$	6.275$\cdot 10^{-5}$	4.125$\cdot 10^{-5}$	6.6$\cdot 10^{-5}$
	$\hbox {Persons}^{-1}\cdot$ $\hbox {Days}^{-1}$	3.3$\cdot 10^{-4}$	8.5$\cdot 10^{-5}$	6.275$\cdot 10^{-5}$	4.125$\cdot 10^{-5}$	6.6$\cdot 10^{-5}$
	$\hbox {Days}^{-1}$	1/24	1/24	1/24	1/24	1/24
	$\hbox {Days}^{-1}$	1/6	1/6	1/6	1/6	1/6
	$\hbox {Days}^{-1}$	1/160	1/160	1/160	1/160	1/160
${\overline{\nu }}_s$	$\hbox {km}^{2}\cdot$ $\hbox {Persons}^{-1}\cdot$ $\hbox {Days}^{-1}$	4.35$\cdot 10^{-2}$	1.98$\cdot 10^{-2}$	0.9$\cdot 10^{-2}$	0.75$\cdot 10^{-2}$	2.175$\cdot 10^{-2}$
${\overline{\nu }}_e$	$\hbox {km}^{2}\cdot$ $\hbox {Persons}^{-1}\cdot$ $\hbox {Days}^{-1}$	4.35$\cdot 10^{-2}$	1.98$\cdot 10^{-2}$	0.9$\cdot 10^{-2}$	0.75$\cdot 10^{-2}$	2.175$\cdot 10^{-2}$
${\overline{\nu }}_i$	$\hbox {km}^{2}\cdot$ $\hbox {Persons}^{-1}\cdot$ $\hbox {Days}^{-1}$	1.0$\cdot 10^{-4}$	1.0$\cdot 10^{-4}$	1.0$\cdot 10^{-4}$	1.0$\cdot 10^{-4}$	1.0$\cdot 10^{-4}$
${\overline{\nu }}_r$	$\hbox {km}^{2}\cdot$ $\hbox {Persons}^{-1}\cdot$ $\hbox {Days}^{-1}$	4.35$\cdot 10^{-2}$	1.98$\cdot 10^{-2}$	0.9$\cdot 10^{-2}$	0.75$\cdot 10^{-2}$	2.175$\cdot 10^{-2}$
$\overline{A}$	Persons	1.0$\cdot 10^{3}$	1.0$\cdot 10^{3}$	1.0$\cdot 10^{3}$	1.0$\cdot 10^{3}$	1.0$\cdot 10^{3}$

The values change with date as these correspond to various restrictions (or relaxtions) taken by the government during the epidemic. We note that these parameters are not normalized in space

**Fig. 13:**
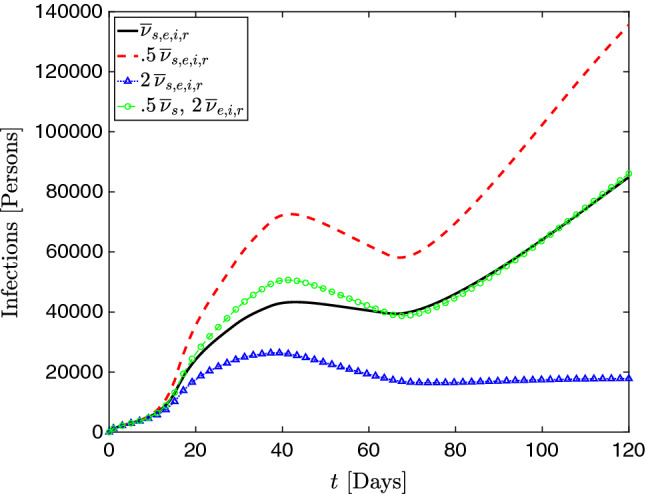
Time evolution of the total active infections *I*(*t*) throughout the entire region of Lombardy, showing that diffusion has a strong influence on the dynamics of disease infection. The double-diffusion case has a distinctly different qualitative pattern, with no substantial increase after $t=60$, while the baseline and half-diffusion cases increase significantly. The dynamics of *I*(*t*) for the case in which ${\overline{\nu }}_s$ is halved while ${\overline{\nu }}_r$, ${\overline{\nu }}_e$, and ${\overline{\nu }}_i$ are doubled suggests that varying each of these diffusion parameters may induce dramatically different changes in the evolution of the outbreak. In the particular scenario considered here, the number of total infected cases grows slighlty faster and has a higher peak when compared to the baseline case

In Fig. 13, we plot the time evolution of the total active infections *I*(*t*) throughout the entire region of Lombardy. Both the baseline and half-diffusion simulations show a distinct long-term growth trend that is not observed in the double-diffusion case. While it is tempting to say that increased diffusion leads to reduced outbreak severity, the reality is more complex. Indeed, the case in which ${\overline{\nu }}_s$ is halved while ${\overline{\nu }}_r$, ${\overline{\nu }}_e$, and ${\overline{\nu }}_i$ are doubled shows a higher peak and slightly faster growth when compared to the baseline simulation, although the long-term growth more closely resembles the baseline than either the double-diffusion and half-diffusion cases. This makes intuitive sense, as the low diffusion among the susceptible population leads to higher population densities and more contagion, while increasing diffusivity among the exposed and infected compartments accelerates the speed and area of propagation. As discussed in [22], the spatial pattern predicted by the heterogeneous diffusion shows generally good agreement with reality; however, nonlocal transmission is not possible using the model given by Eqs. 39-43 and the addition of nonlocal operators, such as fractional diffusion operators [27], is an area for future development.

**Fig. 14:**
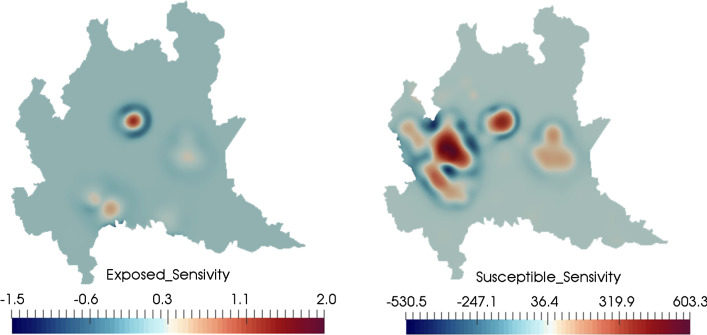
Sensitivity of the computed baseline solution at $t=20$ days for sensitivity to ${\overline{\nu }}_e$ (left) and ${\overline{\nu }}_s$ (right). The sensitivity to ${\overline{\nu }}_e$ is based primarily on currently affected regions, reflecting the state of epidemic progression. The sensitivity to ${\overline{\nu }}_s$, corresponds primarily to highly populated regions. Even though the number of exposed and infected patients is low in certain heavily populated regions (particularly the area around Milan, in the west), the high susceptible sensitivity shown here indicates the region’s vulnerability to the pandemic (which does eventually occur)

In Fig. 14, we plot the computed sensitivity parameters found using Eq. (45) at $t=20$ days. The shown plots quantify sensitivity to ${\overline{\nu }}_e$ (left) and ${\overline{\nu }}_s$ (right). The regions most sensitive to ${\overline{\nu }}_e$ are regions currently affected by the outbreak. However, the sensitivity to ${\overline{\nu }}_s$ shows larger values in more highly-populated areas. At the time shown, the area around Milan (in the west of the shown region, the most populated area of Lombardy) was not experiencing a large outbreak in cases. This is reflected in its relatively low sensitivity to ${\overline{\nu }}_e$. However, its high sensitivity to ${\overline{\nu }}_s$ indicates its vulnerability, irrespective of its current outbreak status. Indeed, the Milan area was ultimately heavily impacted by the epidemic [28].

**Fig. 15:**
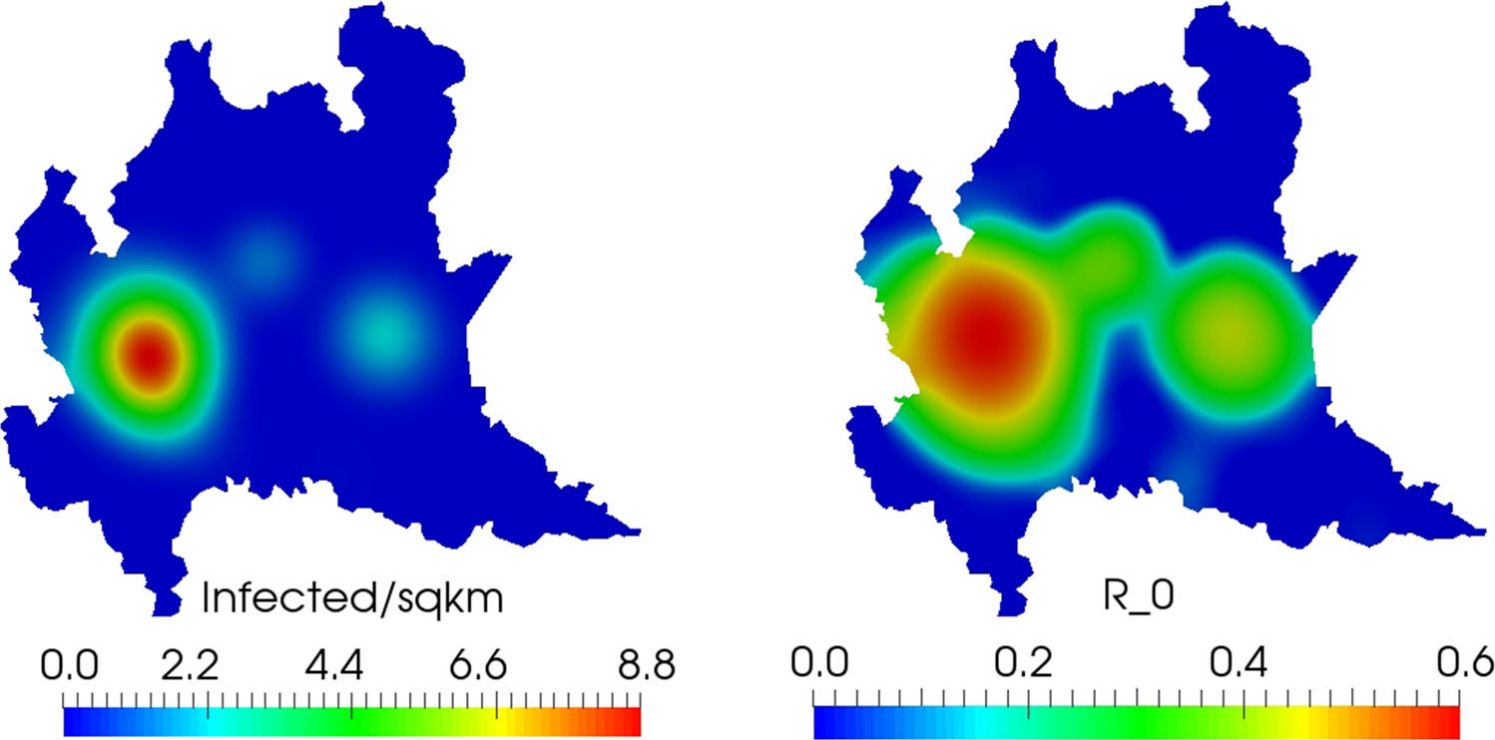
Comparison between $R_0$ value and infected population. Even though $R_0<1$ globally, we still observe growth in some regions, suggesting that the definition (69) of $R_0$ does always not hold in the presence of diffusion

Finally, we find the definition of $R_0$ given by Eq. (69) to be less useful as a predictor of disease spread than in the 1D simulation. This is likely due to the increased role of diffusion. We observe increase in disease exposure and infected individuals in areas where $R_0<1$ both locally and globally, particularly around Milan (as shown in Fig. 15). This suggests the need to revise the definition of $R_0$ in Eq. (69) for the PDE version of the model to account for the influence of diffusion.

## Conclusions

In this work, we introduced a new notational framework for understanding reaction–diffusion compartmental models by interpreting them as balance equations similar to those found in continuum mechanics. We first used this system to derive and explain a simple two-compartment Lotka–Volterra model as a simple example. We then examined a more complex compartmental system: the model of COVID-19 spatiotemporal contagion dynamics introduced in [22]. We showed that this model may be regarded as a sort of conservation law, further justifying the continuum-mechanics type interpretation.

We proceeded to formally derive the model’s sensitivity to diffusion, describe its growth and decay, and establish its stability in the $L^1$ norm. We then looked at an ODE version of the model, using it to derive a basic reproduction number $R_0$ as well as analyzing its spectrum. Additionally, we performed a series of numerical simulations, showcasing the role that numerical methods, diffusion, and $R_0$ play in the behavior of the system. We found that implicit models are effective in describing the temporal dynamics of the system, and second-order in-time methods in particular. We also found that the ODE-based $R_0$ is not consistently reliable as applied to the PDE model, as it worked well for the 1D simulations but did not for the corresponding 2D simulations.

For future work on the COVID-19 model, we would like to extend the diffusion to model the effects of geographic features like roads, rivers, and mountains. We would also like to examine the effectiveness of the model over larger geometries and longer time intervals against measured data. To render the model more effective to decision-makers, incorporating an age-structured population is important for accurately evaluating aspects such as hospitalizations and mortality. The model may also be extended to account for the effects of vaccination on adults, by introducing movement between the susceptible and recovered population [1]. More generally, we would like to apply the continuum mechanics framework shown here to a larger class of compartmental models. In particular, in the field of mathematical epidemiology alone, there are many variants of the SIR-models shown here. For instance, the framework established in the present work may be used for *susceptible-infected-susceptible* models (such as those used for the common cold), or *Maternal-Susceptible-Exposed-Infected-Recovered* models in which immunity is inherited from the mother [1, 5, 20].

## Footnotes

1 There is nothing special or physical about the choice of species. Unfortunately, as ‘predator’ and ‘prey’ both begin with the same letter, a generic notation would be, in the author’s opinion, more arbitrary and confusing.

2 Note: For the discussed model, we have two compartments in two dimensions, giving $\varvec{u}$ dimension 2 $\times$ 1. For this model, the physics is sufficiently simple such that one may define $\varvec{E}$ simply as a 2 $\times$ 2 matrix. However, in general, *E* may be a higher-order tensor if one wishes to define the $\nu$ as tensor quantities rather than the scalar quantities used here.
